# Editorial: Epigenetic Regulation and Tumor Immunotherapy

**DOI:** 10.3389/fonc.2022.893157

**Published:** 2022-05-05

**Authors:** Hongyin Sun, Bihui Huang, Jian Cao, Qin Yan, Mingzhu Yin

**Affiliations:** ^1^ Department of Dermatology, Hunan Engineering Research Center of Skin Health and Disease, Hunan Key Laboratory of Skin Cancer and Psoriasis, Xiangya Hospital, Central South University, Changsha, China; ^2^ Scientific Research Center, The Seventh Affiliated Hospital, Sun Yat-sen University, Shenzhen, China; ^3^ Department of Medicine, Robert Wood Johnson Medical School, Cancer Institute of New Jersey Rutgers, the State University of New Jersey, New Brunswick, NJ, United States; ^4^ Department of Pathology, Yale School of Medicine, New Haven, CT, United States

**Keywords:** epigenetic regulation, tumor immunotherapy, tumor immune microenvironment, methlation, acetylation

Epigenetics is the study of DNA sequence-independent heritable changes in phenotype and gene expression. Major epigenetic mechanisms include DNA methylation, histone modification, chromatin structure regulation, and non-coding RNA regulation. Emerging evidence suggests that epigenetic regulation plays central roles in tumor immunosurveillance, including tumor antigen production, the interaction between tumor cells and immune cells, and T cell development, priming, activation, and exhaustion. On the other hand, tumors commonly hijack various epigenetic mechanisms to escape immune restriction (1, 2). Therefore, modulating epigenetic regulators can normalize the impaired immunosurveillance and/or trigger antitumor immune responses. Numerous preclinical and clinical studies revealed that epigenetic agents, including DNMT inhibitors (3), HDAC inhibitors (4), EZH2 inhibitors (5), LSD1 inhibitors (6), KDM5 inhibitors (7), G9a inhibitors (8), and BET inhibitors (9–11), have the capacity to induce anti-tumor immune responses and modulate tumor immune microenvironment. Currently, hundreds of clinical trials that combine epigenetic agents and immune checkpoint inhibitors (ICI) are ongoing, aiming to achieve synergistic effects, reduce adverse effects, and overcome intrinsic and acquired resistance. Thus, combining epigenetic therapy with immunotherapy is a promising new strategy to improve clinical outcomes.

This Research Topic leads to a better understanding of epigenetics in tumor immunity and immunotherapy and highlights the clinical significance of epigenetic drugs. This Research Topic accepted a total of 18 articles from 123 authors, demonstrating great interest in this field. Our topic can be mainly divided into the following topics:

## Methylation

Abnormal methylation patterns of tumor cells are mainly manifested in the overall hypomethylation of the genome and hypermethylation of CpG islands. The hypermethylation in the promoter regions of tumor suppressor genes and DNA repair leads to the extinction of these genes and the development of cancer. Nevertheless, the hypomethylation in the regulatory regions of oncogenes increases their expression and leads to tumorigenesis (12). In this Research Topic, Zheng et al. explored whether epigenetic regulation associated with DNA methylation could underlie increasing PD-L1 expression by disulfiram (DSF). They found that DSF inhibited DNMT1 expression and activity, thus leading to IRF7 hypomethylation and PD-L1 upregulation in Triple Negative Breast Cancer (TNBC) cell lines. They further observed that co-treatment of DSF and anti-PD-1 Ab increased CD8+ tumor-infiltrating lymphocytes (TIL) and enhanced the therapeutic effects of ICB *in vivo*, which provide a novel combination therapy strategy for TNBC. Based on patients’ overall survival (OS), Yang et al. Established a prognostic risk score system using 18 immune-related methylation genes (IRMGs) of 1057 breast cancer patients from the TCGA cohort and GSE72308 cohort. Patients in the low-risk group had a higher immune score and stromal score compared with the high-risk group. The characteristics based on 18-IRMGs signature were related to the tumor immune microenvironment and affected the abundance of tumor-infiltrating immune cells. As the result, the proposed 18-IRMGs signature could be a potential marker for breast cancer prognostication. Enhancer of zeste homolog 2 (EZH2) is a negative regulator of early NK cell differentiation and function through trimethylation of histone H3 lysine 27 (H3K27me3). Yu et al. deleted *Ezh2* from immature NK cells and downstream progeny to explore its role in NK cell maturation by single-cell RNA sequencing. They indicated a novel role for the EZH2-AP-1-KLRG1 axis in altering the NK cell maturation trajectory and NK cell-mediated cytotoxicity, which suggested that EZH2 plays a critical role in NK development by activating AP-1 family gene expression independent of its methyltransferase activity.

Different from DNA methylation, RNA methylation modifications, including N6-methyladenosine (m6A), 5-methylcytosine (m5C), and N1-methyladenosine (m1A), mainly regulate genetic expression at the post-transcriptional level (13, 14). Liu et al. comprehensively assessed N1-methyladenosine (m1A) methylation modification patterns in 474 ovarian cancer (OC) patients and linked them to immune infiltration characteristics in the tumor microenvironment (TME). They demonstrated that individual tumor m1A modification patterns can predict patient survival, stage and grade. A high m1Ascore is usually accompanied by a better survival advantage and a lower mutational load. Patients with high m1Ascore showed marked therapeutic benefits and clinical outcomes in terms of chemotherapy and immunotherapy, which provide clinicians with new ideas for immuno-oncology and individualized immunotherapy in OC. Recently, m6A RNA methylation is an emerging epigenetic modification, which has been associated with the progression of several cancers (15, 16). ALKBH5 and YTHDF1 are regarded as the eraser and reader in N6-methyladenosine (m6A) modification, respectively. The former has been shown to regulate suppressive immune cell accumulation in melanoma (17, 18), and the latter can improve the efficiency of mRNA translation (19). Using consensus clustering based on the expression of ALKBH5 and YTHDF1, Yan et al. divided the patients with colon adenocarcinoma (COAD) into two clusters. Cluster 2 (high expression of ALKBH5 and lesser so of YTHDF1) had stronger immune infiltration, higher expression of targets of ICIs, more TMB, and a larger proportion of deficiency in mismatch repair-microsatellite instability-high (dMMR-MSI-H) status than Cluster 1 (high expression of YTHDF1 and lesser so of ALKBH5). ALKBH5 and YTHDF1 influence immune contexture and can potentially transform cold tumors into hot tumors in patients with COAD. In addition, m6A modification accelerates Snail1 expression in HeLa cells (20), indicating the indirect regulation of Snail1 by methylation. Snail1, a key inducer of epithelial-mesenchymal transition (EMT), plays a critical role in tumor metastasis. Tang et al. reviewed the pathways and molecules involved in the maintenance of Snail1 level and the significance of Snail1 in tumor immune evasion and demonstrated that Snail1 can function as a biomarker to predict tumor relapse and patient prognosis. Furthermore, Snail1 is implicated in chemotherapy and radiotherapy resistance, thereby the author proposes that chemotherapy or radiotherapy combined with Snail1 inhibitors may be a promising therapeutic approach to combat tumors.

## Acetylation

Acetylation is the addition of acetyl groups to lysine residues in a protein that occurs in the presence of acetyltransferase, which is a dynamic and reversible process involving both histone acetyltransferases (HATs) and histone deacetylases (HDACs). HATs are figuratively called “writers”, which are responsible for covalently attaching an acetyl group to the lysine residue of a protein, while HDACs are called “erasers” and mediate the removal of this acetyl group (21). Evidence has shown that acetylation is one of the most important modifications used to alter protein activity and precisely regulate and control cellular functions. In recent studies, many researchers have found that HDAC inhibitors also have significant effects on host immunosuppressive cells, and MDSCs are important immunosuppressive cells in the tumor microenvironment (22). Thereby some researchers regard MDSCs as targets of tumor therapy. Cui et al. summarized the effects and the underlying mechanisms of different HDAC inhibitors on the immunosuppressive function and expansion of MDSCs based on the findings of relevant studies, which may improve their therapeutic effects on tumors.

## Long Non-Coding RNA Regulation

Long non-coding RNA (lncRNA) has been reported to play diverse roles in various biological processes (23), which can modulate transcriptional and post-transcriptional genes and regulate the expression of tumor suppressors or initiators, and thereby confers the occurrence and progression of cancer (24). In this topic, Xu et al. constructed a novel hypoxia-related long non-coding RNAs (HRL) signature that could distinguish lower-grade glioma (LGG) patients with similar expression levels of immune checkpoints and might predict the efficacy of immune checkpoint inhibitors. Additionally, hypoxia-related pathways and immune pathways were enriched in the high-risk group, and a high risk score indicated low tumor purity and high immune infiltration. LINC00941 and BASP1-AS1 could significantly affect the proliferation of glioma cells. These results reveal that HRLs could be novel biomarkers to predict the prognosis of LGG patients and potential targets for LGG treatment. Simultaneously, Yu et al. built a prognostic model signature based on six key immune-related lncRNAs (irlncRNAs) (H19, ST3GAL6-AS1, AL162231.2, SOX21-AS1, AC006213.5, and AC002456.1) in glioblastoma multiforme (GBM) patients. PLAU was predicted as a target of lncRNA-H19 and mainly enriched in the malignant related pathways. Moreover, three hub genes KRT8, NGFR, and TCEA3 among GBM subtypes (GSs) were screened and validated to potentially play oncogenic roles in GBM. These results suggested that the irlncRNAs had promising potential for immunotherapy of GBM.

In addition to the above three topics, Ma et al. found that SAMD9 (Sterile Alpha Motif Domain-Containing Protein 9) was highly expressed in glioma and closely related to histological and genetic features in CGGA and TCGA databases. They present evidence to show that there was a positive association between SAMD9 and malignancy characters in LGG. Immune infiltration analysis demonstrated that high SAMD9 expression resulted in an accumulation of macrophages by CIBERSORT and TIMER databases, the same trends were also verified using clinical specimens with IHC staining. In addition, silencing of SAMD9 by shRNA in LN229 cells attenuated the infiltration abilities of M2 macrophage. Taken together, the authors revealed that SAMD9 may be a diagnostic or prognostic indicator for LGG and also a new potential therapeutic target for treating gliomas. Moreover, Xu et al. identified IFN-γ response clusters, which might be used to improve the prognostic accuracy of immune contexture in the clear cell renal cell carcinoma (ccRCC) microenvironment. Immune-cold RANBP2-type and C3HC4-type zinc finger containing 1 (RBCK1)^high^ patients have pro-tumorigenic immune infiltration and significantly worse outcomes than RBCK1^low^ patients based on results from multi-omics to real-world data, which highlights the association between tumor alterations and immune phenotype. Furthermore, Zhou and Jin presented the expression and biological function of B7 homolog 3 protein (B7-H3) in distinct cancer and normal cells, as well as B7-H3-mediated signal pathways in cancer cells and B7-H3-based tumor immunotherapy strategies, which provides a comprehensive overview that encompasses B7-H3’s role in TME to its potential as a target in cancer immunotherapy.

## Conclusion

In aggregate, this Research Topic summarized recent development on the central roles of epigenetic regulation in tumor immunosurveillance and the mechanisms of epigenetic regulation. This topic provides new prognostic biomarkers, as well as promising therapeutic approaches and novel combination therapy strategies for several tumors.

## Author Contributions

All authors listed have made a substantial, direct, and intellectual contribution to the work and approved it for publication.

## Conflict of Interest

The authors declare that the research was conducted in the absence of any commercial or financial relationships that could be construed as a potential conflict of interest.

## Publisher’s Note

All claims expressed in this article are solely those of the authors and do not necessarily represent those of their affiliated organizations, or those of the publisher, the editors and the reviewers. Any product that may be evaluated in this article, or claim that may be made by its manufacturer, is not guaranteed or endorsed by the publisher.
